# A smartphone microscopic method for simultaneous detection of (oo)cysts of *Cryptosporidium* and *Giardia*

**DOI:** 10.1371/journal.pntd.0008560

**Published:** 2020-09-08

**Authors:** Retina Shrestha, Rojina Duwal, Sajeev Wagle, Samiksha Pokhrel, Basant Giri, Bhanu Bhakta Neupane

**Affiliations:** 1 Center for analytical sciences, Kathmandu Institute of Applied Sciences, Kathmandu, Nepal; 2 Department of Environmental Science, Tri-Chandra Multiple Campus, Tribhuvan University, Kathmandu, Nepal; 3 Central Department of Chemistry, Tribhuvan University, Kathmandu, Nepal; Foundation for Innovative New Diagnostics (FIND), SWITZERLAND

## Abstract

**Background:**

Food and water-borne illness caused by ingestion of (oo)cysts of *Cryptosporidium* and *Giardia* is one of the major health problems globally. Several methods are available to detect *Giardia* cyst and *Cryptosporidium* oocyst in food and water. Most of the available methods require a good laboratory facility and well-trained manpower and are therefore costly. There is a need of affordable and reliable method that can be easily implemented in resource limited settings.

**Methodology/Principle findings:**

We developed a smartphone based microscopic assay method to screen (oo)cysts of *Cryptosporidium* and *Giardia* contamination of vegetable and water samples. The method consisting of a ball lens of 1 mm diameter, white LED as illumination source and Lugols's iodine staining provided magnification and contrast capable of distinguishing (oo)cysts of *Cryptosporidium* and *Giardia*. The analytical performance of the method was tested by spike recovery experiments. The spike recovery experiments performed on cabbage, carrot, cucumber, radish, tomatoes, and water resulted in 26.8±10.3, 40.1±8.5, 44.4±7.3, 47.6±11.3, 49.2 ±10.9, and 30.2±7.9% recovery for *Cryptosporidium*, respectively and 10.2±4.0, 14.1±7.3, 24.2±12.1, 23.2±13.7, 17.1±13.9, and 37.6±2.4% recovery for *Giardia*, respectively. The spike recovery results are comparable with data obtained using commercial brightfield and fluorescence microscope methods. Finally, we tested the smartphone microscope system for detecting (oo)cysts on 7 types of vegetable (n = 196) and river water (n = 18) samples. Forty-two percent vegetable and thirty-nine percent water samples were found to be contaminated with *Cryptosporidium oocyst*. Similarly, thirty-one percent vegetable and thirty-three percent water samples were contaminated with *Giardia cyst*.

**Conclusions:**

The newly developed smartphone microscopic method showed comparable performance to commercial microscopic methods. The new method can be a low-cost and easy to implement alternative method for simultaneous detection of (oo)cysts in vegetable and water samples in resource limited settings.

## Author summary

Food and water-borne illness arising from the consumption of contaminated food and water are serious health hazards globally. *Cryptosporidium* and *Giardia* are the major food and water‒borne parasites. The infection occurs mainly by (oo)cyst phase of the parasites. People in developing countries are more vulnerable to these parasites where infection is more likely underdiagnosed and underreported due to limited resources for detection. There is need of a method that is affordable and easy to implement. In this study, we developed and optimized a novel smartphone microscope method that can detect and quantify the (oo)cyst of the parasites in food and water samples. The developed method is easy to implement and affordable and provides similar performance to the other commercially available microscopic methods.

## Introduction

Food and water-borne illness arising from the consumption of contaminated food and water are serious health hazards globally [1]. The World Health Organization (WHO) has reported 1.5 billion episodes of diarrhoeal cases leading to 3.5 million deaths of under 5-year-old children in developing countries annually. More than 70% of these diarrhoeal episodes are attributable to biologically contaminated food [2]. In order to prevent and identify the disease, detection of food and water borne parasites is important at all levels of production chain followed by screening and certification [3].

*Cryptosporidium* and *Giardia* are the major food and water-borne parasites [4]. Ninety percent of reported outbreaks of these pathogenic protozoans occur through water, while 10% are related to food. In the infective stage, *Cryptosporidium* oocysts have spherical shape with a diameter of 4–6 μm and *Giardia* cysts have elliptical shape of 8–12 um long and 7–10 μm wide [5]. Both of the cysts, collectively termed as (oo)cysts, have a simple and direct life cycle, which is extremely suitable for transmission by fresh produce. Additionally, the cysts are small in size with a robust transmission stage. Some genotypes of the parasites even have zoonotic potential giving the opportunity for contamination to occur from both animal and human sources. *Cryptosporidium* are particularly threatening as they are resistant to chlorine disinfection, can persist in the environment for a long period, can infect other animal hosts, and are difficult to diagnose and treat. The infectious dose for *Giardia* cysts and *Cryptosporidium* oocysts are 10‒100 and 10‒1000 respectively, which makes these pathogens more precarious [6]. Developing countries are the most vulnerable countries to these protozoans where infection is more likely underdiagnosed and underreported, and has limited resources for investigation [7]. In low income countries, the overall prevalence rate of *Giardia* infection is 20–30% and the occurrence of *Cryptosporidium* is 4‒31% in children younger than 10 years [8].

Several highly sensitive and specific methods have been described to detect *Giardia* cyst and *Cryptosporidium* oocyst in food, water, and faecal samples. Commonly used approaches are polymerase chain reaction, flow cytometry, optical microscopic examination etc. However, these techniques need a good laboratory facility, well trained user and are expensive, therefore are not appropriate for low‒resource settings including remote and field sites. There is a need for a simple, easy to use, rapid but reliable and low‒cost test method for the detection of parasites [9–12].

In recent years, smartphone based systems are being explored and used as an alternative platform for the detection of microscopic to sub‒microscopic specimens and parasites [13] in a wide variety of matrices, such as parasite eggs in faecal sample[14], allergen in food [15], blood cells in blood [16], single nanoparticles and viruses [17], filarial and malarial parasites in blood [18, 19], sickle cell anaemia in a blood smear [20], soil‒transmitted helminth and fluke in urine and stool samples [21] etc.

In this work, we describe a smartphone microscopic system that can image and quantify (oo)cysts of both *Cryptosporidium* and *Giardia* in a given sample. We optimized and measured optical parameters of the microscope including field of view, magnification, and image contrast under different staining and illumination conditions. The validity of the developed microscope was tested by spiking the vegetable and water samples with known number of standard (oo)cysts samples. For comparison, the spiked samples were also imaged with a commercial bright field and a fluorescence microscope and the percentage recovery data were compared. The optimized smartphone microscope was then used to measure (oo)cyst contamination in un-spiked vegetable and water samples.

## Materials and methods

### Design of smartphone microscope

**Fig 1:**
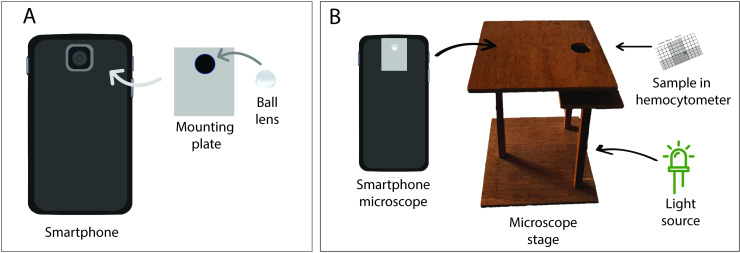
Components of smartphone microscope. (A) Ball lens mounted onto the mounting plate and then to the smartphone camera. (B) Measurement set up.

We used a sapphire ball lens (Edmund Optics, New Jersey, USA) and an aluminium mounting plate to transform a smartphone (Samsung Galaxy J7 prime) into a smartphone microscope. A small hole was punctured at the centre of the mounting plate and the ball lens was firmly glued in this hole (Fig 1A). The ball lens was then centred over the smartphone camera lens. The mounting plate was fixed onto the smartphone using a transparent tape. The smartphone had a rare camera of 13 MP and the screen size of the phone was 151.7 mm x 75 mm with 1080 x 1920 pixels resolution. We custom built a microscope stage to hold the sample slide using a wooden viewing box of dimension 15×15 cm. A 3 cm diameter hole was drilled on top centre position of the box to have the illumination light pass through the sample specimen placed on the microscopic slide just above the hole. The slide was fixed on both sides of the hole using a double-sided tape. A schematic of the microscope set up is shown in Fig 1.

### Measurement of magnification and contrast

We tested three ball lenses having different diameters of 0.5 mm, 1 mm, and 2 mm. Image of a standard calibration grid (Generic, USA; 1 division = 10μm) was captured with the ball lens attached to the smartphone. The distance covered by all the grid lines was measured in pixel using ImageJ software. The pixel distance was converted to micrometre to get field of view (FOV) of the microscopic system.

The magnification (*MAG*) was calculated as:

$$MAG=\frac{b}{a}$$ [1]

Where, *a* and *b* are size of object and image, respectively.

To measure the contrast, we imaged standard (oo)cysts sample (Waterborne, Inc., New Orleans, USA) under different illumination and staining conditions to determine the image contrast. Based on field of view and magnification, we chose 1 mm ball lens in our further experiments. We tested two different light sources for sample illumination: a smartphone flashlight and a white light emitting diode (LED, 12 watt). We also tested two different types of stains: Lugol’s iodine (HiMedia, Mumbai, India) and methylene blue dye (Fisher Scientific, New Hampshire, United States). The staining experiments involved a mixture of *Cryptosporidium parvum* and *Giardia intestinalis* (oo)cysts suspension (~ 2×10^5^ (oo) cyst per mL) and stain dye in 1:1 ratio. An aliquot of 10 μL of the mixture was loaded on the Hemocytometer (Max Levy, Philadelphia, USA) and was then illuminated by the light source and viewed through smartphone microscope. For each light source, images of (oo)cysts were captured at different time intervals after mixing the dye with the standard (oo)cysts. The images were analysed in ImageJ software to calculate the contrast in percentage (C) as follows [22]:

$$C=\left(\frac{I_{\max}-I_{\min}}{I_{\max}+I_{\min}}\right)100$$ [2]

where, *I*_*max*_ is the maximum intensity on the specimen of interest i.e. (oo)cyst and *I*_*min*_ is the average intensity of immediately adjacent background.

### Spike recovery experiments

Five different types of vegetables such as tomato, cabbage, carrot, radish, and cucumber were selected for spike recovery experiments. These vegetables were selected based on previous reports of faecal contamination [23–26]. These vegetables are consumed in raw forms in many countries. All the samples were bought from the local vegetable shops. A portion of the sample (15–20 g) was soaked in distilled water for about 20 minutes to remove all the surface contaminants including (oo)cysts. After washing the samples, 10 μL of a mixture of *C*. *parvum* oocysts and *G*. *intestinalis* cyst suspension (Waterborne Inc, PC101 G/C positive control) was added to randomly selected points on sample surface samples with a micropipette. The (oo)cysts seeded sample was left to dry at room temperature for 2 hours to overnight.

We also performed similar spike recovery experiments with water samples. Five sets of 50 mL distilled water (pH = 6.8, conductivity = 0.05 μS/cm) were spiked with 10 μL of the standard (oo)cyst suspension and were incubated overnight.

We tested three different washing solutions such as distilled water, normal saline, and glycine buffer (1M, pH 5.5) to extract (oo)cysts from each spiked vegetable samples. The sample was put in extracting solution in a ziplock bag (Great Value, Fresh Seal Double Zipper). Ziplock bag was used as a low‒cost alternative to stomacher bag. The eluate was then carefully transferred into two 50 mL falcon tubes. The elute suspension was concentrated by using the triple centrifugation method proposed by Medeiros and Daniel [27] with some modifications. At first eighty millilitres of eluate was centrifuged in two 50 mL tubes at 1500 x g for 10 minutes. The supernatant was decanted into a clean beaker leaving a final volume of 5 mL, which was placed in a vortex mixture for 20 seconds to homogenize the pellet. The 5 mL residual volume from each centrifugation tubes were combined together into a single tube. Another centrifugation was carried out at 1500 x g for 10 minutes. The supernatant was discarded leaving 0.5 mL pellet in the centrifuge tube. The residual solution was again vortexed for 20 seconds and it was carefully transferred to 1.5 mL microcentrifuge tube with 10 μL micropipette. The centrifuge tube was rinsed with 0.5 mL distilled water and added to the same 1.5 mL microcentrifuge tube to make the final volume of 1 mL. Now, the third centrifugation was performed at 1500 x g for 10 minutes. The supernatant was removed, leaving just 0.5 mL in the microcentrifugation tube one more time.

The water samples containing (oo)cysts were subjected to flocculation and sedimentation as described by Karanis and Kimura [28] with some modifications. 50 mL of ferric sulphate (0.25 M) solution was added to 50 mL of water samples and the pH was adjusted to 6±0.05. The sample was left 24 hours at room temperature to precipitate floc. Then the supernatant was carefully aspirated with a syringe filter without disturbing the sediment. The sediment was further centrifuged at 2,000 × *g* for 10 minutes and the supernatant was discarded. The pellet was dissolved in 1 mL of citric acid lysis buffer (8.4 g citric acid monohydrate, 17.64 g tri‒sodium‒citrate‒dihydrate, distilled H_2_O up to 100 mL; pH 4.7) by incubating at room temperature for 1 hour with vortexing every 15 minutes. The sample was washed twice with distilled water by centrifugation at 2000 ×*g* for 10 minutes. The pellets collected were resuspended in 5 mL distilled water for the purification of (oo)cyts. The purification step is only required with contaminated water, whereas non‒contaminated water can be pelleted followed by dissolving in the buffer and subjected to the microscopy.

The purification steps of water samples involved a discontinuous sucrose gradient. The gradient was prepared with the Sheather's solution (320 mL H_2_O and 500 g sucrose) diluted with 0.025 M phosphate-buffered saline (PBS) and supplemented with 1% Tween 80 to make 1:2 solution of 1.103 specific gravity and 1:4 solution of 1.064 specific gravity. 10 mL of 1:4 solution was layered over 10 mL of 1:2 solution on a 50 mL centrifuge tube. Then, 5 mL of the sample were layered over 1:4 solution and was centrifuged at 1500 × *g* for 30 minutes. The three layers were recovered carefully and pooled separately along with the pellet and examined for the oocysts. The pooled layers were diluted with water, centrifuged and pellets were collected for the microscopic analysis.

### Microscopic measurements

Ten microliters of each concentrated sample were stained with 10 μL of diluted Lugol's iodine (1:2 in water) and subsequently loaded into hemocytometer. The sample was incubated for 6 minutes. The (oo)cysts were screened and enumerated in four quadrants of the hemocytometer under smartphone microscope. The cysts on the same hemocytometer were simultaneously counted by brightfield microscope (#T490B-10MT, 40X‒2000X Trinocular, Amscope, USA). Triplicate measurement was made for each concentrated suspension.

The spiked samples were also examined with a fluorescent microscope (Labomed Inc, United States, LB 702). For fluorescence measurement, 5μL of (oo)cyst suspension was placed on the clean glass slide to which a drop of fluorescein‒labelled mouse monoclonal antibodies (Aqua-Glo G/C direct, Waterborne Inc., USA) was applied. The slide was incubated at 37°C for 10 minutes in an incubator (Faithful, China) and imaged with 480 nm excitation and 520 nm emission wavelengths.

**Fig 2:**
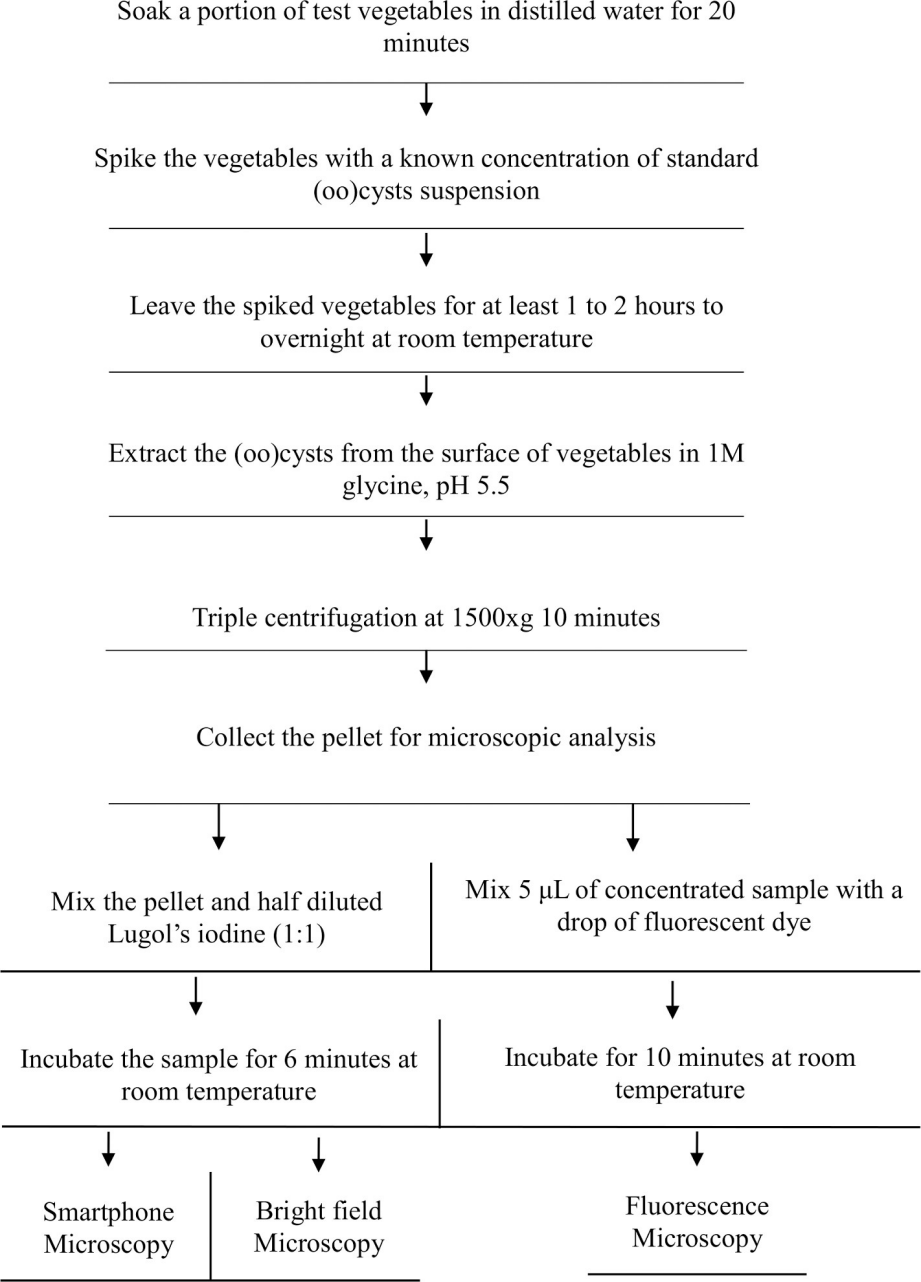
A flow chart describing different steps involved in the spike recovery experiment.

A flow chart summarizing the major steps involved in the spike recovery experiment is shown in Fig 2.

The numbers of (oo)cyst were counted before each seeded experiment using all three microscopic methods. The mean percentage recovery efficiency (RE) was calculated as:

$$RE=\left(\frac{n}{N}\right)100$$ [3]

Where, *N* is the number of (oo)cysts added to a sample and *n* is the number of (oo)cysts recovered from the sample.

It is to be noted that the recovery of (oo)cyst depends on several parameters such as the size/weight and type of sample, interfering particles, and volume of elution buffer. It has been reported that the recovery efficiency increases with smaller sample size, lower volume of elution buffer and minimum level of interfering particles. These conditions reduce the potential losses of (oo)cyst during the sample processing. To obtain a better limit of detection, higher number of (oo)cysts should be spiked for larger amount of sample to start with. Considering all these issues sample size of 15–20 g was used in the recovery experiment. Similar sample weight was used in literature for the recovery experiments of (oo)cysts on vegetable samples by microscopic methods [29, 30]. The % recovery data were reported as the average of 9 measurements (triplicate independent samples and triplicate measurement for each sample) for each type of vegetable and water samples.

We followed the above procedure excluding the washing of raw vegetables and spiking with (oo)cyst to determine the (oo)cyst contamination in the vegetable samples collected from local market. The un-spiked vegetable samples were bought (~500 g) from randomly selected ten different fruits and vegetable markets of Kathmandu, Nepal. The samples were 28 cabbage, 10 carrots, 37 cucumber, 40 green chilli, 10 radish, 31 spinach and 40 tomato (total n = 196). The sample types were chosen based on previous reports of the fecal contamination. The vegetable samples were proceeded according to Environmental Protection Agency method 1623 and *Codex Alimentarius* CAC/GL 33 (FAO, 1999). The vegetable samples were transported in a cooler box to the laboratory for further analysis. Each type of vegetable from the particular vendor was carefully placed on a tray and mixed properly. Then the quartering sampling procedure was applied as a size reduction process to make the vegetable samples statistically representative. Three replicates of each vegetable were selected randomly and sealed in a zip lock bag and stored at 4°C to 10°C at the refrigerator until further use. The tray was wiped with 70% ethanol each time before starting with another set of vegetables to remove the cross-contamination.

### Analytical performance of the method

The analytical parameters such as limit of detection (LOD), sensitivity, specificity, positive predictive values (PPV), and negative predictive values (NPV) were used to check the analytical performance of the smartphone method.

To calculate the limit of detection (LOD), initially, 10 μL of standard (oo)cysts suspension (Waterborne TM, Inc., New Orleans, LA, USA) containing 100 (oo)cysts was used as the stock solution. Then the suspension was serially diluted with distilled water. Each diluted suspension was mixed with Lugol's iodine and numbers of oocysts were measured. For each suspension, triplicate measurement was made. The (oo)cysts were enumerated in four quadrants of the hemocytometer under the smartphone microscope. This process was repeated for all the diluted suspension until at least one (oo)cyst was detected per quadrant. Further, the minimum number of (oo)cysts required in the sample to identify the sample as positive (at least 1 oocyst) was calculated by using recovery efficiency. Then, the calculated minimum number of (oo)cysts is divided by the average weight of the sample used in recovery experiment to derive the minimum number of (oo)cysts per gram required to determine the sample as positive.

Bright field method was used as a reference method to calculate the sensitivity and specificity of the smartphone method. Sensitivity (*Se*) and specificity (*Sp*) was determined as [31]:

$$Se=\left(\frac{TP}{TP+FN}\right)$$ [4]

$$Sp=\left(\frac{TN}{TN+FP}\right)$$ [5]

Where, *TP* (true positive) are the positive samples detected by both smartphone microscope and the reference method, *TN* (true negative) are samples detected negative by both the microscopes, *FP* (false positive) are the sample detected positive only by the smartphone microscope method, and *FN* (false negative) are the samples detected only by the reference method.

The positive predictive value (PPV) is the probability of the presence of contamination given in a positive test result whereas negative predictive value **(**NPV) is the probability of the absence of contamination given in a negative test result. These parameters were calculated as follows [31]:

$$PPV=\frac{Se.Pl}{Se.Pl+(1-Sp)(1-Pl)}$$ [6]

$$NPV=\frac{Sp(1-Pl)}{\left(1-Se)Pl+Sp(1-Pl\right)}$$ [7]

where, prevalence (*Pl*) is defined as total number of positive samples/ total number of samples. Kappa (κ) statistic was calculated to assess the diagnostic agreement between the methods, given by [32, 33]:

$$\kappa =\frac{OA-EA}{1-EA}$$ [8]

Where, OA and EA are the observed and expected agreements and defined as follows.

$$OA=\frac{TP+TN}{n}$$ [9]

$$EA=\frac{\left(TP+FP)(TP+FN)+(TP+FP)(TP+FN\right)}{n^{2}}$$ [10]

κ>0.8 signify almost perfect agreement, values between 0.6 and 0.8 indicate substantial agreement, values between 0.4 and 0.6 show slight to the moderate agreement, and values between 0.2 and 0.4 indicate a fair agreement.

### Statistical analysis

Data were organized in a spreadsheet (Microsoft Excel, 2020) and analysed by using descriptive statistics. The Quantile-Quantile (Q-Q) plot was used to check if the data are distributed on both sides of the mean. For normally distributed data, two tail paired *t*-test was used to check the level of significance between two related samples for different variables. A *p* value less than 0.05 was considered as significant.

## Results and discussion

### Optimization of smartphone microscope

The performance of an imaging system is determined by its optical parameters, such as field of view, magnification, resolution, and contrast. The FOV is the size of the viewing area that can be seen when we look through a microscope. Magnification measures the zooming of an object, and resolution and contrast measure the details and clarity in an image [34]. The FOV of ball lens based imaging system depends on the size of ball lens, the refractive index of ball lens material and wavelength of illumination source; the size factor being major contributor [35]. On the other hand, the magnification of smartphone microscope depends on size of ball lens and also on the nature of smartphone that contains a built-in lens and CMOS camera at fixed distance. The spherical ball lens has a curved surface that results in curvature effect. It means the central region is more sharp/clear than the periphery in the image plane. The clear field of view microscope having ball lens of 0.5, 1, and 2 mm ball lens is provided in Table 1.

**Table 1:** Field of view (FOV) of the smartphone microscope.

Diameter of ball lens (mm)	Clear field of view (μm)
0.5	114±6
1	203±6
2	490±10

The measured field of view (FOV) of smartphone microscope showed that the FOV increases with an increase in the diameter of ball lens (Table 1) which is in consistent with reported values [35].

The magnification of smartphone microscope with 1 mm ball lens was estimated to be 200×. The 1 mm lens set up was able to magnify *Cryptosporidium* oocyst to 0.8−1.2 mm and *Giardia* cyst to 1.4−2.4 mm, respectively. The magnification was enough to distinguish the two specimens. Therefore, we selected a smartphone microscopic system having 1 mm diameter ball lens for further experiments in this study.

**Fig 3:**
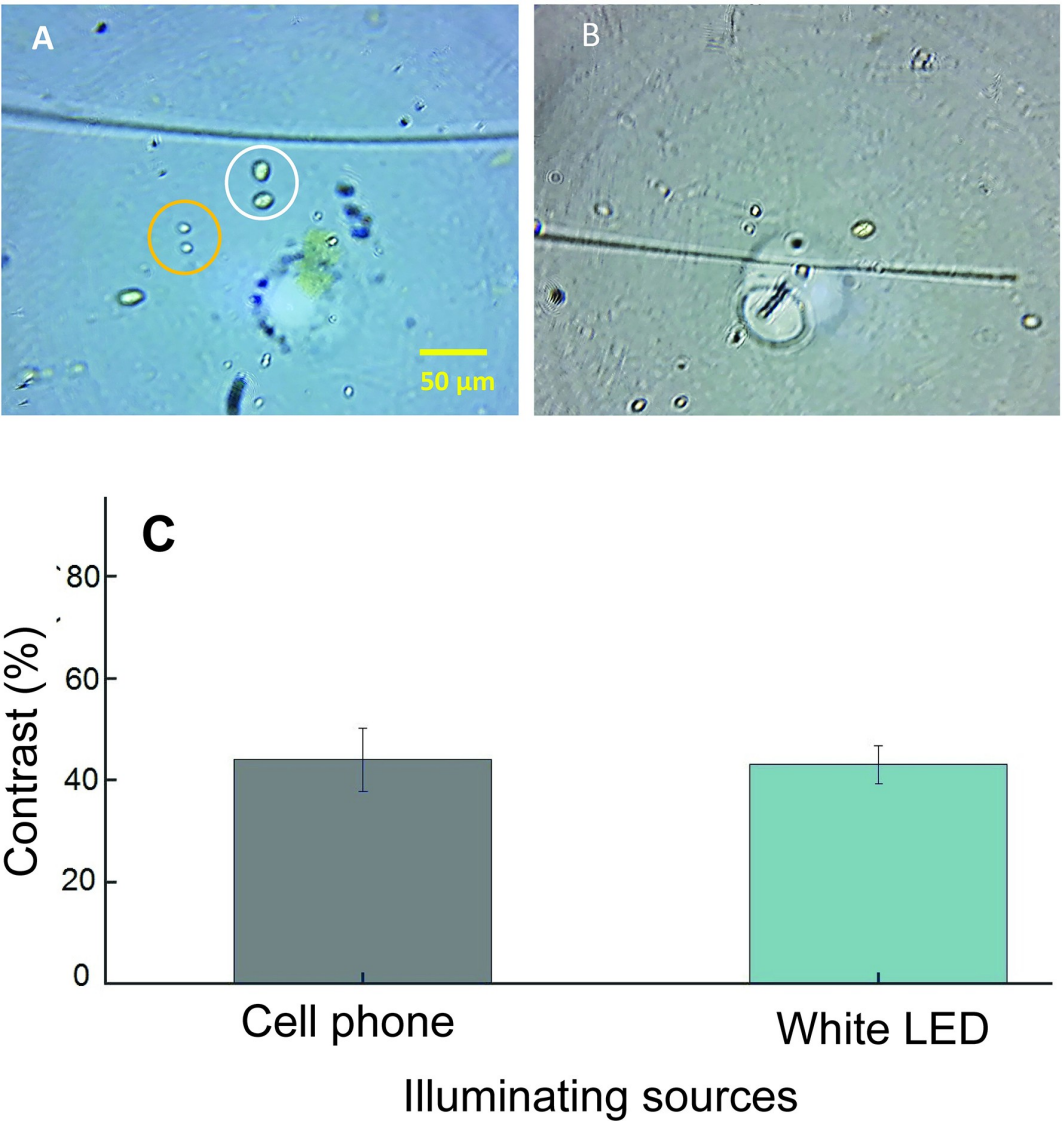
Images and data obtained with different illumination sources. (A) Representative images of (oo)cysts taken using smartphone microscope with 1 mm ball lens with white LED light illumination and (B) smartphone flashlight illumination. The representative cyst and oocyst in A are shown within white and yellow circles, respectively. A scale bar of 50 μm is shown in A and also applies for B. (C) The measured contrast for (oo)cysts. The error bar in C represent the standard deviation of triplicate measurements.

Light source used for illuminating sample affects the image quality of (oo)cyst. We tested following two types of commonly available light sources: white LED and smartphone light. The images collected using these light sources are shown in Fig 3.

Images shown in Fig 3 clearly depict that the oocyst and cyst can be easily distinguished from each other based on their shape and size. Oocysts are circular in shape and cysts are oval. The contrast for the (oo)cysts is shown in Fig 3C. Both of the light sources provided similar contrast percentages. Since the white LED is easily available, cheaper, and easy to use, we chose it for further experiments.

**Fig 4:**
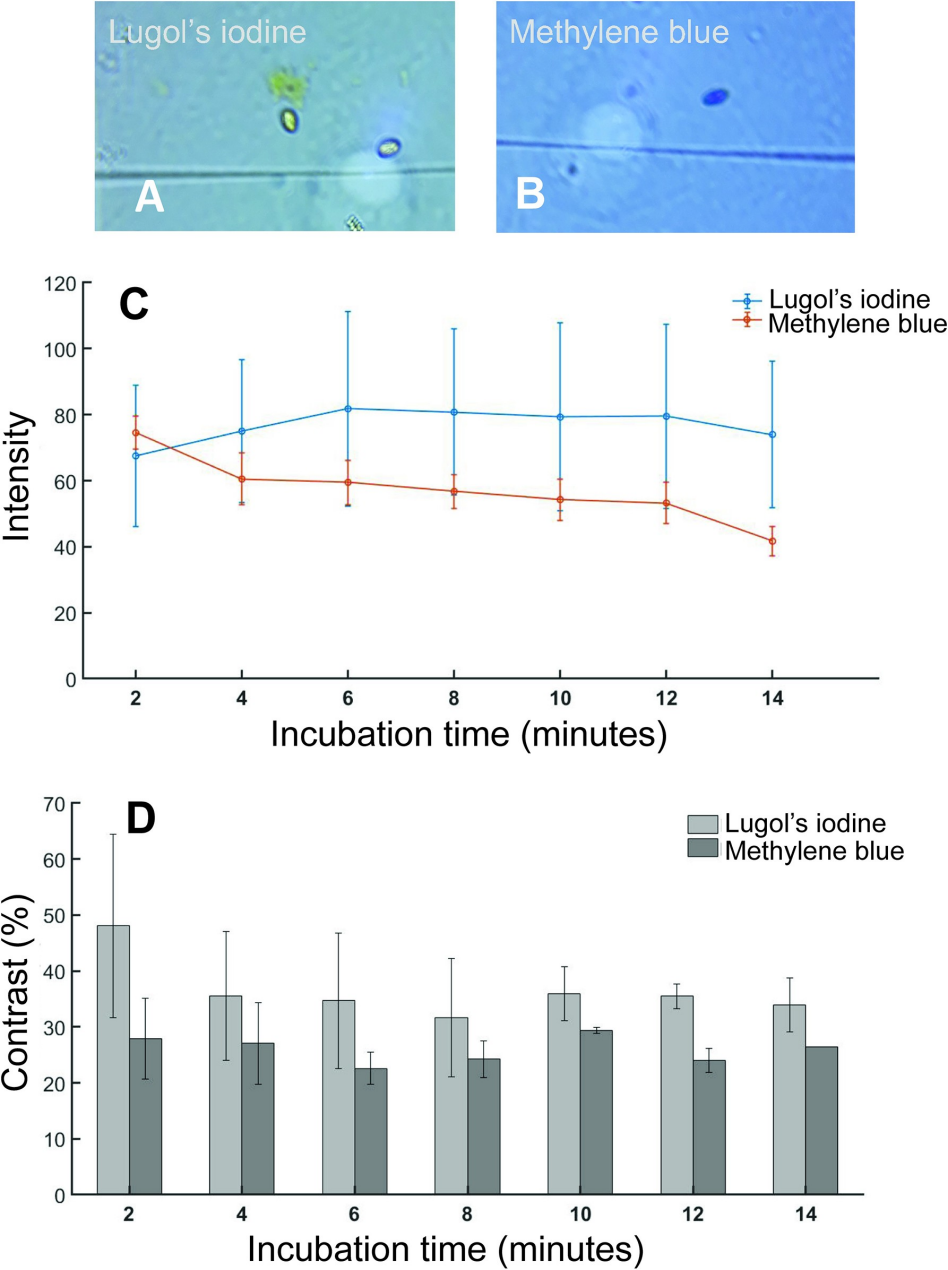
Images and data obtained with two different staining methods. (A) Representative images of Lugol’s iodine and (B) methylene blue staining. The images were taken at 10 min of staining. (C) The average intensity of (oo)cysts under white LED light source at different time intervals (D) A plot of contrast versus incubation time for methylene blue and Lugol’s iodine staining.

A number of staining procedures have been developed to aid in the clear morphological identification and differentiation of (oo)cysts by light microscopy [9]. Some of the most used techniques are the iodine and methylene blue mounts. These methods are simple, faster and inexpensive and provide clear distinction of (oo)cyst by morphological features [10]. The temporal variation of stain color intensity on the cysts are shown in Fig 4C. This shows the color intake by the cysts and stability of the stains with waiting time.

It is evident that Luglo’s iodine (LI) provided brighter image than Methylene blue (MB). Also, LI staining, intensity increased after 2 minutes of incubation and remained constant for up to 12 minutes (Fig 4C). This indicated that LI staining is more stable over time. Based on stability of stain and intensity, we selected LI staining and 6–10 minutes of staining time in the subsequent experiments. The Lugol’s iodine staining also provided higher contrast compared to methylene blue dye staining. Lugol’s iodine initially has contrast of 48±16.4% which decreased to 35.5±11.6% in next 2 minutes and remained constant throughout the time (Fig 4D). In case of methylene blue contrast remained constant around 27.7% during the experiment.

### Method validation

Accuracy of the smartphone microscope was evaluated by spike recovery experiments using both vegetable and water samples. In this experiment, known number of (oo)cyst were spiked to the sample and the number of (oo)cyst recovered were counted with the smartphone microscope. We compared the results from smartphone microscope with measurements using commercial bright field and fluorescence microscopes (Table 2).

**Table 2:** Percentage recovery of *Cryptosporidium* and *Giardia* using smartphone, commercial brightfield, and fluorescence microscopes.

Sample	Recovery (%)					
	Smartphone microscope		Brightfield microscope		Fluorescence microscope	
	Cryptosporidium	Giardia	Cryptosporidium	Giardia	Cryptosporidium	Giardia
Cabbage	26.8±10.3	10.2±4.0	40.2±7.1	21.6±6.7	21.2±3.2	18.4±2.6
Carrot	40.1±8.5	14.1±7.3	62.7±15.8	24.7±11.4	22.8±5.5	20.1±6.3
Cucumber	44.4±7.3	24.2±12.1	51.9±18.3	26.7±10.6	22.8±3.5	19.9±4.1
Radish	47.6±11.3	23.2±13.7	60.3±12.3	26.7±11.9	23.9±5.0	20.4±2.9
Tomato	49.2±10.9	17.1±13.9	58.3±14.3	21.7±9.0%	32.3±6.6	29.2±2.8
Water	30.2±7.9	37.6±2.4	35.3±7.0	45.6±6.6	44.4±8.8	55.8±7.4

The recovery of *Giardia* ranged from 10.2±4.0% in cabbage to 37.6±2.4% in water and recovery of *Cryptosporidium* ranged from 26.8±10.3% in cabbage to 49.2±10.9% in tomato using smartphone microscope measurement (*see* Table 2). For most of the samples, the percentage recovery was found to be higher in bright field microscopy than in smartphone microscopy (paired t-test, *p*<0.05). The recovery of oocyst was higher than the cyst in all three microscopes with few exceptions such as recovery in cucumber and tomato by fluorescence no significant difference was observed (*p*>0.05). In image plane (in camera), the smartphone microscope has circular field of view having diameter of ~200 μm, whereas the commercial bight filed microscope at 400× has rectangular field of view of ~190 μm×350 μm. The lower percentage recovery in smartphone than in brightfield microscope may have arisen due to lower field of view which makes (oo)cyst counting difficult.

The recovery efficiencies also varied in certain percentages among all five different vegetables within the same method. In smartphone microscopy, the recovery efficiency of cysts of *Giardia* in radish is significantly greater than that of carrot and cabbage (paired t-test, *p*<0.05), while it is comparable for pair of other vegetables. In the case of *Cryptosporidium* oocysts, cabbage had a significantly lower recovery in comparison to carrot, tomato and radish (P<0.05). While the bright-field microscopy had a significantly higher recovery of oocysts in carrot, tomato, and radish than in cabbage, but no significant difference was observed for cyst in all the vegetables. The fluorescence method had a significantly higher recovery percentage for both *Giardia* and *Cryptosporidium* in tomato than other vegetables (paired t-test, *p*<0.05). The difference in recovery efficiencies in various vegetables using the same methodology might be due to the variability of the noncovalent interactions between (oo) cyst surfaces and surfaces of various vegetables we tested. It is also important to note that the extraction methods that have been proven suitable for one specific food matrix could be unsuitable for the others [36–39]. For example, the glycine wash buffer had satisfactorily recovered both *Giardia* and *Cryptosporidium* in lettuce and raspberries [14, 36–38]. On the other hand, a prolonged, vigorous washing of *Spinacia oleracea* [40] and apples [41] in 1 M glycine (pH 5.5) elution buffer was not able to remove all of the *Cryptosporidium* oocysts from their matrix[40, 41].

The recovery using fluorescence microscope, in which the (oo)cysts were tagged with fluorescent dye tagged antibody, was found to be lower than in remaining two microscopes, except in water samples. Fluorescence microscopy is dependent on binding of the fluorescence tagged antibodies to the antigen surface, which can be hindered/altered by the impurities present in the solution like the vegetable debris in our case. In some cases, fluorescent antibody could bind to the impurities and show the false positive. This could be an explanation for the higher fluorescence in the sample purified from the tomatoes. In addition of hindering binding of antibodies to oo(cysts), the larger particles can deposit (oo)cysts underneath so that they are no more accessible for antibodies. So, lower detection in case of radish, cabbage, cucumber and carrot could be due to decreased binding affinity of the antibody to the (oo)cysts or hiding of (oo) cysts underneath the larger vegetable debris [43].

The percentage recovery data reported in this work are comparable to literature studies. Cook *et al*. [42] reported the percentage recovery of *Giardia* and *Cryptosporidium* in spiked lettuce and raspberries of 30.4% and 44.3%, respectively. In another study, Cook *et al*., [37] developed a method for simultaneous detection of *Giardia* cysts and *Cryptosporidium* oocysts on lettuces and other salad products. The immunomagnetic separation and texas red staining resulted in *Giardia* cyst and *Cryptosporidium* recoveries on a variety of commercially available natural foods of 36.5±14.4% and 36.2 ±19.7% (n = 20) respectively. Similarly, in a study conducted by Amoro *et al*. [26] in 19 salad products, following the same method of Cook *et al*. [36, 37, 42], recoveries of the texas red–stained *Cryptosporidium* and *Giardia* were 24.5± 3.5% and 16.7 ±8.1% respectively.

Table 2 also lists the recovery efficiencies of the spiked water samples, detected by all three microscopic methods. The recovery of both *Giardia* cysts (55.9±7.4%) and *Cryptosporidium* oocysts (44.4±8.8%) were higher in fluorescence microscope (paired t-test, P<0.05) compared to both smartphone and bright field microscopes. For smartphone microscope, 37.6±2.4% cysts and 30.2%±7.9% oocysts were observed whereas it was 45.6±6.6% cysts and 35.3±7.0% oocysts in bright field microscopy. Previous studies have reported similar percentage recovery data. Le Chevallier et al. used the immunofluorescence microscopic method and reported an average recovery efficiency of 68.6% for *Giardia* cysts and 25.3% for *Cryptosporidium* oocysts in seeded tap water [44]. In another study Le Chvallier et al. showed a recovery of 96% and 77% for cysts and oocysts respectively, with the Percoll sucrose density gradient at a specific gravity ≥ 1.10 [45]. Koompapong et al. using a similar methodology reported a recovery of oocysts (75%) in water samples [46]. In contrast, Machado et al. found a significantly small recovery of 5.3%, who analyzed the sediment of water samples using Kinyoun and Koster histochemical staining techniques [47]. They didn’t use any chemical precipitant for the flocculation of oocysts before purification steps. Karanis et al (2001) compared different flocculants and concluded that using ferric sulfate yield a higher recovery (61.5%) of *C*. *parvum* oocysts from tap water with a very low impact on the viability of oocysts [28]. Also, no detergent solutions were included in the study that helped to set the oocysts free from the sediments [47]. In a study made by Hsu et al. standard Envirochek capsule filtration followed by immunomagnetic separation, the standard purification procedure in Environmental Protection Agency Method 1623, was used. In their study, the recovery efficiencies were higher for *Giardia* (48.0%) than for *Cryptosporidium* (32%) [48]. These data are very similar to our percentage recovery data in water samples.

We also estimated method detection limit (LOD) of smartphone microscope method. The LOD varied with type of sample. LOD of *Giardia* ranged from 24 cyst/100 g for cucumber to 73 cyst/100 g for cabbage (tomato = 38, carrot = 40 and cucumber = 23 cyst/100 g). Similarly, the LOD for *Cryptosporidium* ranged from 11 oocyst/100 g for radish to 25 oocyst/100 g for cabbage (tomato = 12, carrot = 12 and cucumber = 23 oocyst/100 g). In general, the LOD of *Cryptosporidium* was lower than that of *Giardia*.

### Prevalence of (oo)cysts in vegetable and water samples

**Fig 5:**
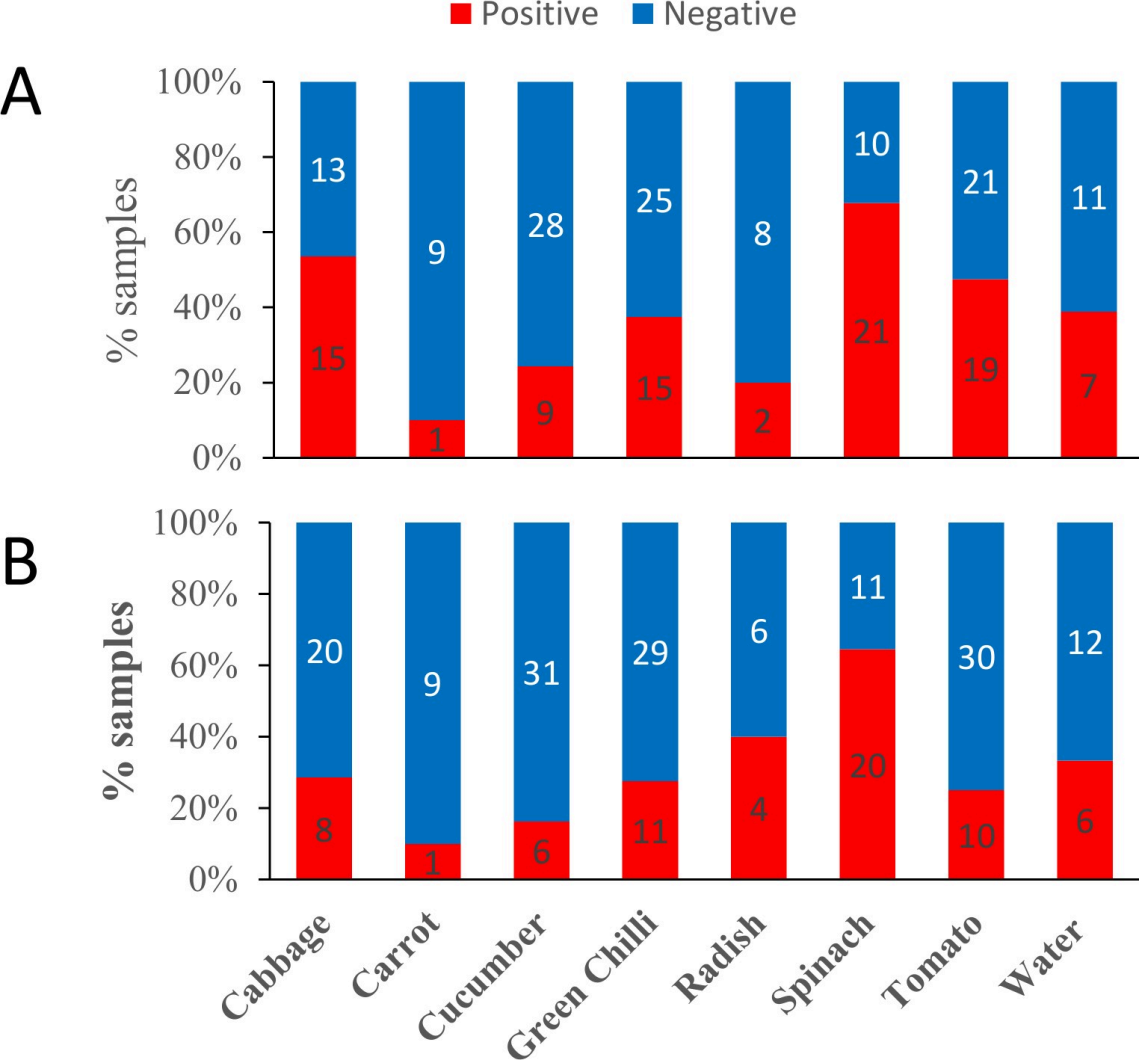
Prevalence of *Giardia* and *Cryptosporidium* in vegetable and water samples measured by smartphone microscopic method. A) Cryptosporidium and B) Giardia. The numbers within each bar graph represent the number of samples.

After developing the smartphone microscopic system for (oo)cyst detection, we screened (oo)cysts contamination in five different types of vegetable samples purchased from local market in Kathmandu, Nepal. The sample analysed were 28 cabbage, 10 carrots, 37 cucumber, 40 green chilli, 10 radish, 31 spinach and 40 tomato (total n = 196). We also screened the (oo)cysts contamination in 18 river water samples. All the samples were also analysed by bright field microscopy. Further, out of 196 vegetable samples, randomly selected 58 (30%) samples were screened using fluorescence microscope. The samples were processed and analysed as described in method section. The prevalence data for oocysts and cysts in different samples as measured by smartphone microscopy method is shown in Fig 5A and 5B, respectively. Among the vegetables, the highest prevalence was found in spinach samples and lowest in carrot samples. The difference in prevalence might arise due to difference in the shape and local surface properties of vegetables. The (oo)cysts can easily attach to the uneven or curly surfaces of spinach and cabbage either in the farm or when washed with polluted water. On the other hand, vegetables with smooth surfaces such as radishes and carrots had a low number of (oo)cysts in the present study as its smooth surface reduces the attachment of the protozoans [43].

We also segregated the number of samples that contained either cyst or oocyst (singular) or both cyst and oocyst (mixed) in the all the sample types. The data for all microscopic methods is summarized in Table 3.

**Table 3:** Singular and mixed prevalence of (oo)cysts in vegetable and water samples measured by three different microscopic methods.

*Sample type*	*Result type*	*Smartphone* *Microscopy*			*Bright field* *Microscopy*			*Fluorescence* *Microscopy*		
		C*	G*	*both*	C*	G*	*both*	C*	G*	*both*
*Cabbage*	*positive*	*6*	*0*	*9*	*1*	*4*	*8*	*4*	*0*	*4*
	*negative*	*13*	*19*	*13*	*19*	*16*	*15*	*1*	*5*	*1*
*Carrot*	*positive*	*0*	*0*	*1*	*0*	*0*	*1*	*0*	*0*	*1*
	*negative*	*9*	*9*	*9*	*9*	*9*	*9*	*0*	*0*	*0*
*Cucumber*	*positive*	*3*	*0*	*6*	*0*	*3*	*6*	*3*	*1*	*3*
	*negative*	*28*	*31*	*28*	*31*	*28*	*28*	*3*	*5*	*2*
*Green Chilli*	*positive*	*5*	*1*	*10*	*3*	*2*	*12*	*3*	*0*	*2*
	*negative*	*25*	*29*	*24*	*25*	*26*	*23*	*6*	*9*	*5*
*Radish*	*positive*	*0*	*2*	*2*	*0*	*1*	*2*	*0*	*3*	*0*
	*negative*	*8*	*6*	*6*	*8*	*7*	*7*	*3*	*0*	*0*
*Spinach*	*positive*	*2*	*1*	*19*	*1*	*2*	*19*	*3*	*5*	*4*
	*negative*	*10*	*11*	*9*	*11*	*10*	*9*	*10*	*8*	*5*
*Tomato*	*positive*	*11*	*2*	*8*	*1*	*5*	*15*	*1*	*5*	*0*
	*negative*	*21*	*30*	*19*	*24*	*20*	*19*	*9*	*5*	*3*
*Water*	*positive*	*7*	*6*	*0*	*6*	*2*	*0*	*4*	*4*	*0*
	*negative*	*11*	*2*	*5*	*12*	*16*	*10*	*14*	*14*	*10*

C* *= Cryptosporidium only*

G* *= Giardia only*

In a total sample of 196 vegetables, 3.1% of samples (6/196) contained *Giardia* (only), 13.8% samples (27/196) contained *Cryptosporidium* (only), and 28.1% (55/196) contained both (oo)cysts when detected with a smartphone microscope. To compare the results, we also tested the samples with the brightfield microscope and the fluorescence microscope. 8.7% of samples (17/196) were positive for singular cysts, 3.1% vegetable samples (6/196) were positive for singular oocysts and both (oo) cysts were positive in 32.1% samples (63/196) using the brightfield microscope. In the fluorescence microscopy, out of 58 randomly selected samples, 24.1% of samples (14/58) detected cyst (only), 24.1% of samples (14/58) detected oocyst (only), and 24.1% of samples (14/58) detected both (oo) cysts.

According to a survey conducted by Maikai *et al*. 40% of Spinach, 32% of tomato, 24% of carrot and 16% of cabbage were contaminated with *Cryptosporidium* oocysts as determined by microscopy [50]. A study by Kaudah *et al*. reported that 50% tomatoes, 43.1% cabbage and 26.4% of carrot tested positive for different protozoans among which 11% were *Cryptosporidium*. These samples were stained with Lugol’s iodine and observed under light and fluorescence microscope [51]. In contrast Utaaker *et al*. reported that only 14% (8/56) tomatoes and 9% (4/47) cabbages were contaminated with either *Giardia* cysts or *Cryptosporidium* oocysts [23]. In case of root vegetables such as carrot, the current study has a very low record (10%) for both *Giardia* and *Cryptosporidium*. Similar results were also reported in other studies—14% positive cases in India [23] and 6.4% in Southern Ethiopia [52] for *Giardia*. A slightly higher positive cases was observed in Egypt (43.3%) [49] and Korea (33.3%).

We also estimated the (oo)cysts per unit of sample. The highest concentration of the (oo)cysts for both *Giardia* and *Cryptosporidium* were detected in cabbages (n = 28) with the concentration of 442 cysts and 225 oocysts/kg. The lowest concentration of 35 cysts/kg and 16 oocysts/kg was found in radish. Tomatoes (n = 40), carrots (n = 10), and cucumbers (n = 37) were found to be contaminated with 129 cysts/kg, 166 cysts/kg, and, 77 cysts/kg, respectively. Similarly, 76 oocysts/kg, 47 oocysts/kg, 185 oocysts/kg, and were found in tomato (n = 40), carrots (n = 10), and cucumber (n = 37), samples. The infectious dose for cryptosporidiosis and giardiasis is as low as 10‒30 viable (oo)cyst [53]. Assuming around 200 g of poorly washed raw vegetable is consumed per day, there is still high chance that most of (oo)cyst containing sample could be infectious if ingested.

We tested eighteen surface water samples collected from 3 different sites of the Bishnumati river, Kathmandu, Nepal in two different field campaigns. The samples were flocculated and purified with sucrose density gradient and examined by smartphone, commercial bright field, and fluorescence microscopies. A total of 33.3% (6 out of 18 samples) were positive for *Giardia* and seven samples (38.9%) were positive for *Cryptosporidium* by smartphone microscope (Table 3). When compared to other microscopes, in general, higher number samples tested positive for (oo)cysts by the smartphone microscopy. Brightfield microscope confirmed 22.2% (4 out of 18) positive results for *Giardia* and 33.3% (6 out of 18) positive results for *Cryptosporidium*. Similarly, four water samples (22.2%) were positive for (oo)cysts by using fluorescence microscopy *(see* Table 3). However, none of the methods detected samples having both cysts and oocysts.

Bright-field microscopy results were considered as the reference method to determine the specificity, sensitivity, PPV, NPV, and kappa (κ) of the smartphone microscopy for the detection of *Giardia* and *Cryptosporidium* in vegetable sample and water samples. In our study, smartphone microscope had 55 (28%), 158 (80%), 11 (5.6%) and 26 (13.2%), and 66 (33%), 153 (78%), 23 (11.7%), and 9(4%) true positive, true negative, false positive and false negative cases for *Giardia* and *Cryptosporidium*, respectively. The smartphone microscopy had a sensitivity, specificity, positive predictive value, and negative predictive value of 67%, 93%, 83%, and 85% for *Giardia* and 88%, 86%, 74%, and 94% for *Cryptosporidium*, respectively. The diagnostic agreement between the smartphone microscope and the bright field microscope was determined based on the calculated κ (kappa) value. The substantial agreement was observed between the microscopes with κ = 0.64 and κ = 0.71 for *Giardia* and *Cryptosporidium*, respectively.

In the present study, (oo)cysts were confirmed and counted manually on the basis of shape, size and contrast. The manual counting is time consuming. In future, the manual counting could be replaced with an automated identification and counting method. The microscopic method described in this paper does not test the viability of (oo)cysts. Viability testing would certainly be interesting to explore in future. The microscopic methods based on morphology and contrast alone do not provide species specific identification of the (oo)cysts. It would also be interesting to make a comparative study on the analytical performance of smartphone method with more established methods such as polymerase chain reaction as a complementary method could that can provide species specific information when needed. To summarize, we designed the smartphone microscope and optimized its various optical parameters. The field of view increases with the diameter of sapphire ball lens but the magnification follows the opposite trend; in agreement with theory. We found that microscope having ball lens of 1 mm diameter along with Lugol's iodine staining and commercially available white LED illumination can simultaneously determine (oo)cyst of *Cryptosporidium* and *Giardia* in vegetable samples. The spiking recovery experiment on the different vegetable and water samples showed that the % recovery is comparable to the commercial bright field microscope and better than fluorescence microscopic measurement. We found that % recovery varied with the nature of sample and recovery for *Cryptosporidium* oocyst is better than *Giardia* cyst. This observation is consistent with the literature studies.

We also used the method to detect and quantify (oo)cyst in different vegetable and water samples. We found that out of the 196 vegetable samples 31.1% vegetable samples were positive for cysts and 42% samples were positive for oocysts contamination when examined by smartphone microscope.

This study shows that the smartphone based microscopic assay can be a low-cost alternative for screening of (oo)cyst of *Cryptosporidium* and *Giardia* in resource limited settings. The approximate cost of our microscope (excluding the cost of smartphone) is ~$15. This method also has the potential to be used in clinical settings. Educational institutions can also adopt this method for teaching and learning objectives. Our future work involves the development of an automated smartphone program that could take image, process the image to identify and count the (oo)cysts, and provide report to the user. This automated system may minimize error and shorten the analysis time.

## Supporting information

S1 Data Raw data for the variation of intensity and contrast with the incubation time. (XLSX)

S2 Data Raw data for spike recovery in vegetable and water samples. (XLSX)
